# Patterns in Age-Seroprevalence Consistent with Acquired Immunity against *Trypanosoma brucei* in Serengeti Lions

**DOI:** 10.1371/journal.pntd.0000347

**Published:** 2008-12-09

**Authors:** Sue Welburn, Kim Picozzi, Paul G. Coleman, Craig Packer

**Affiliations:** 1 Centre for Infectious Disease, College of Medicine and Veterinary Medicine, The University of Edinburgh, Edinburgh, United Kingdom; 2 Department of Infectious and Tropical Diseases, London School of Hygiene and Tropical Medicine, London, United Kingdom; 3 Department of Ecology, Evolution, and Behavior, University of Minnesota, Minnesota, United States of America; Yale University School of Medicine, United States of America

## Abstract

Trypanosomes cause disease in humans and livestock throughout sub-Saharan Africa. Although various species show evidence of clinical tolerance to trypanosomes, until now there has been no evidence of acquired immunity to natural infections. We discovered a distinct peak and decrease in age prevalence of *T. brucei* s.l. infection in wild African lions that is consistent with being driven by an exposure-dependent increase in cross-immunity following infections with the more genetically diverse species, *T. congolense* sensu latu. The causative agent of human sleeping sickness, *T. brucei rhodesiense*, disappears by 6 years of age apparently in response to cross-immunity from other trypanosomes, including the non-pathogenic subspecies, *T. brucei brucei*. These findings may suggest novel pathways for vaccinations against trypanosomiasis despite the notoriously complex antigenic surface proteins in these parasites.

## Author Summary

Trypanosomiasis is a major health threat in Africa, but vaccine development has long been hampered by the extraordinarily diverse surface proteins of these parasites. However, Serengeti lions show an asymptotic age prevalence of the non-pathogenic *Trypanosoma congolense* in contrast to a strong peak and decrease in age prevalence of the pathogenic *T. brucei* s.l.. This pattern suggests that lions may gain cross-immunity to *T. brucei* from repeated exposure to the more genetically diverse *T. congolense*. Although lions may gain more effective cross immunity than other host species owing to their frequent consumption of infected prey animals, these findings suggest possible strategies for designing effective vaccines against sleeping sickness in livestock and humans.

## Introduction

Trypanosomes transmitted by tsetse flies are a major constraint to the health and economic development of many of the poorest regions of Africa. Infections in humans result in over 50,000 deaths each year, while animal trypanosomiasis is one of the most important livestock diseases across Africa [1]. The search for effective vaccines to protect both humans and their livestock populations from the trypanosomiases has proved to be one of the greatest and most elusive challenges in global public health [1]. This is because of the unique mechanism of immune invasion employed by the trypanosomes.

Persistence of trypanosome infection depends on evasion of the host immune response through a complex system of antigenic variation of the variant surface glycoprotein (VSG) that shields the cell [2]. Once host antibodies recognize any one VSG, trypanosomes expressing that VSG are killed. Antigenic variation involves a stochastic switch in the VSG gene expressed from a repertoire of possibly a thousand genes; the switching subset of trypanosomes survives [2]. Although wildlife [3] and cattle [4] show evidence of clinical tolerance to trypanosomes, until now there has been no evidence of acquired immunity to natural infections. The absence of any natural examples of immunity to trypanosomes has been a major constraint to vaccine development.

## Materials and Methods

### Study design

**Figure 1:**
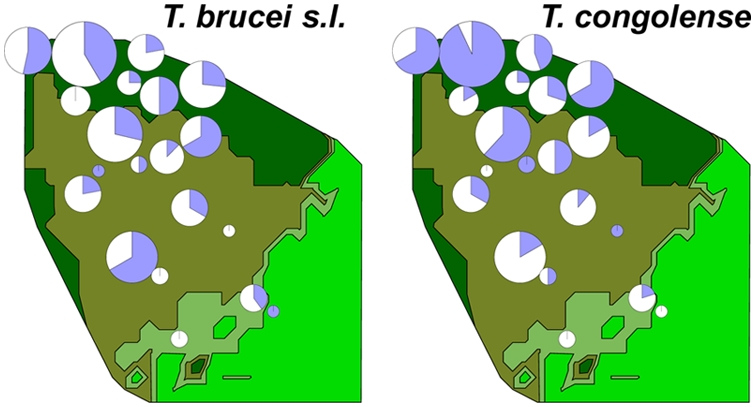
Map of the 2,000 km^2^ Serengeti study area. Map colours indicate habitat: woodlands (dark green), tall-grass plains (olive), intermediate grass plains (pale green) and short grass plains (bright green). Pie-charts show the proportion (in blue) of animals infected with either *T. congolense* or *T. brucei*; size of each chart is proportional to the number of lions sampled in each pride; each chart is centred at the approximate midpoint of each pride's territory.

184 blood samples were taken from 179 Serengeti lions (*Panthera leo*) between 1984 and 1994 as part of long-term ecological and epidemiological studies [5]. The Serengeti lions have been studied continuously since 1966; birthdates (accurate to within +/−1 month) and annual ranging patterns are known for each individual in the study. Most samples were collected during genetic surveys and thus constitute a random sample with respect to health; additional animals were sampled in 1994 during a veterinary surveillance program for canine distemper [6], but these individuals showed no relationship between health and trypanosomiasis. The lion study area comprised two major habitats: 112 of the sampled lions (from which 114 samples were taken) lived in woodlands dominated by *Acacia* and *Commiphora* trees; the remaining 67 lions (from which 70 samples were taken) lived on open grass plains [7] (Fig. 1). Tsetse flies are largely restricted to the woodlands, but large numbers of infected herbivores migrate through both habitats in response to seasonal rainfall.

### Laboratory Procedures

Ethical clearance was granted by the Animal Care committee at the University of Minnesota (reference IACUC 0107A04903), and heparinized blood was collected from each lion, adhering to the institution's guidelines for animal husbandry. Red-cell pellets were frozen at −80°C. For PCR, the red cell pellet was thawed and applied to a DNA binding matrix (Whatman FTA). Single 2-mm blood-saturated discs were cut from the FTA cards to seed each PCR reaction as previously described [8]. Each sample was screened three times for the presence of *T. congolense* s.l. using primers to repeated internally transcribed sequences [9] and for *T. brucei* s.l. using the generic Trypanozoon primers TBR 1 and 2 [10]. Samples that amplified for *T. brucei* were screened five times using primers that amplified the SRA gene specific for human infective *T. b. Rhodesiense* [11].

### Mathematical models

Two mathematical models based on a standard compartmental, linked differential equation framework (see [12] pages 58–86) were fitted to the lion age-prevalence data to examine differences in infections patterns between the two parasite species (*Trypansoma congolense* and *T. brucei s.l.*) in the contrasting transmission-intensity habitats (high-intensity woodlands versus low-intensity plains). For each trypanosome species, both models were fitted to all of the age-prevalence data combined, as well as separately to the plains and woodland data. The age categories for the different habitats are shown in Table 1.

**Table 1:** Details of the age groups used in the mathematical models.

Woodlands		Plains		All	
Average age, years	Number of samples	Average age, years	Number of samples	Average age, years	Number of samples
1.1	22	1.5	14	1.1	26
2.0	22	2.7	14	1.8	26
3.5	22	5.5	14	2.5	26
6.8	22	8.7	14	4.2	26
10.7	26	11.6	14	6.9	26
				8.7	26
				12.0	28
**Total**	**114**		**70**		**184**

#### Model 1: Age-constant force of infection and recovery

The simplest model assumed susceptible lions (*S*) were infected at a rate *λ*, generally referred to as the force of infection which is the per capita rate at which susceptibles acquire infection (see [12] page 51). It was further assumed that *λ* was independent of age (*a*) and infected lions (*I*) recovered from infection at a rate *ν*, upon which they reverted to the susceptible status.

This can be expressed as a set of linked differential equations as
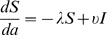
(1)and
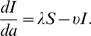
(2)


In this model system, 1/*λ* is the average age of first infection and 1/ν is the average duration of infection.

This model formed the baseline against which the more complicated model incorporating an immune class was compared.

#### Model 2: Immune class

Model 1 was extended by the introduction of an immune class (*R*) following recovery from infection. Immunity was lost at a rate γ (giving an average duration of immunity from infection of 1/γ) with lions reverting to the susceptible status immediately following the loss of immunity.

The linked equations describing the model are:
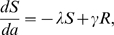
(S3)

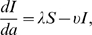
(S4)and
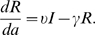
(S5)


All models were fitted to the observed data using standard non-linear maximum likelihood techniques assuming binomial error structure [13]. We tested the statistical significance of moving from the simpler Model 1 to the more complicated Model 2 by comparing the difference in ln-maximum likelihood to the χ^2^ distribution [13]. Model fitting was conducted in Excel.

Survival analysis to examine differences in mortality rates between lions with different trypanosome infection status was conducted using a Cox proportional hazard model with censoring for the six individuals yet to die and controlling for the birth year. The analysis was conducted in Stata.

## Results

**Figure 2:**
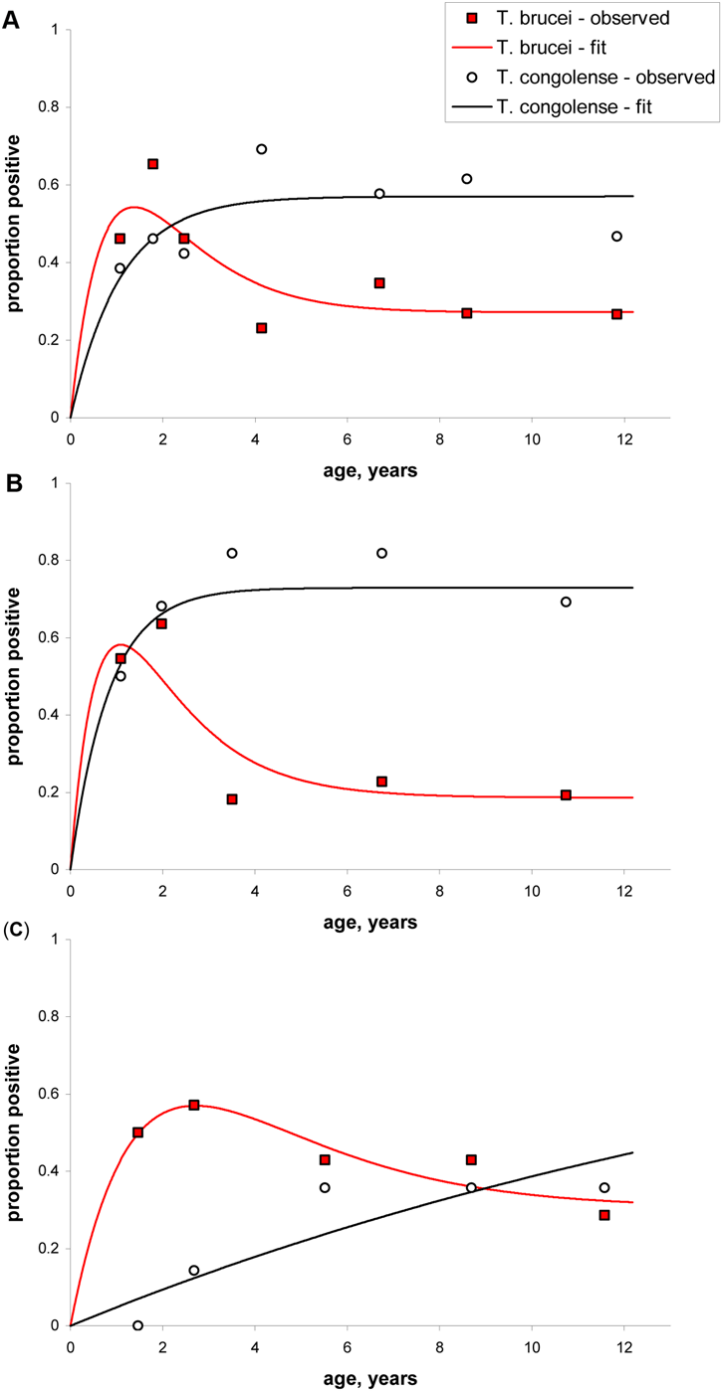
Model fitted to *T. brucei* and *T. congolense* age-prevalence curves in (A) all Serengeti lions, those living in (B) the high tsetse-exposure woodlands and (C) the low tsetse-exposure plains. For the combined data and in both habitats analysed separately, the simple Model 1 assuming a constant force and duration of infection were fitted to the *T. congolense* data and could not be significantly improved by including an immune class. For *T. brucei*, the illustrated model for all three data sets includes a temporary immune class that significantly improves the fit to the data compared to a Model 1 in panels (A) and (B) but was not statistically significant in (C).

The lions show a distinct peak in age prevalence for *T. brucei* s.l., a pattern which was maintained when the data was examined separately for both the plains and woodland habitats, but the pattern in *T. brucei* s.l. age prevalence is statistically more pronounced in woodlands lions living under higher tsetse challenge (Fig. 2B). By contrast, *T. congolense* s.l. infection consistently shows a monotonic increase with age (Fig. 2A, B and C).

**Figure 3:**
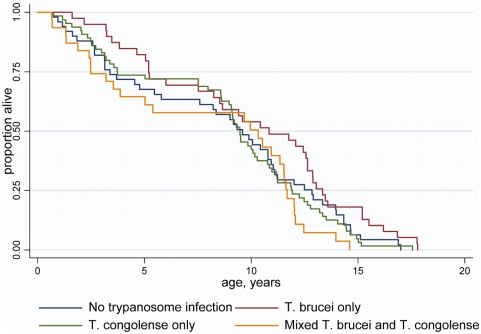
Kaplan-Meier survival curves for Serengeti lions. There was no significant difference in the survival schedules (χ^2^ = 6.76, d.f. = 3, p>0.05, Cox proportional hazard model with censoring for the six individuals yet to die and controlling for the birth year) for lions without any trypanosome infections (median survival time = 9.59 years, 95% CIs 5.79–10.93 years), those infected with *T. brucei* only (10.84, 95% CIs 7.52–12.64), those infected with *T. congolense* only (9.47, 8.61–10.28), and those infected with both species (10.32, 3.54–11.53).

The strong peak in prevalence for *T. brucei* s.l. at 2–3 yrs of age is not due to increased mortality in infected animals, since there was no significant difference in the survival schedules of trypanosome-positive and -negative individuals (Fig. 3). Alternatively, infections would be expected to decline with age with the development of acquired immunity. Using the parameters in Table 2, the simple two-compartment Model 1 was first fitted to the *T. congolense* data, and we then tested whether the addition of some form of temporary protective immunity (Model 2) would significantly improve the fit of the model. For *T. congolense*, Model 1 could not be significantly improved by adopting Model 2 for all the data combined (χ^2^ = 0.850, d.f. = 1, *P* = 0.357) as well as the woodlands (χ^2^ = 0.585, d.f. = 1, *P* = 0.444) and plains (χ^2^ = 0.083, d.f. = 1, *P* = 0.773) data analysed separately (Fig 2A, B and C).

**Table 2:** Model parameters estimates.

Parameter	*T. congolense*			*T. brucei s.l.*		
	All	Woodlands	Plains	All	Woodlands	Plains
1/*λ* years	1.90	1.15	20.26	0.88	0.65	1.62
1/*υ* years	2.52	3.09	648.66	2.10	1.95	4.46
1/*γ* years				4.75	7.86	8.47

Values are shown as the reciprocal of the rate estimate: 1/*λ* is the average age at first infection, 1/*υ* is the average duration of infection and 1/*γ* years is the average duration of immunity.

We then tested whether the *T. brucei* data could be better explained by an acquired immunity that resulted in the establishment of a temporary immune class before reversion to the susceptible class. In contrast to *T. congolense*, inclusion of temporary protective immunity resulted in a statistically significant improvement (χ^2^ = 5.536, d.f. = 1, *P* = 0.019) over the simpler Model 1, when all the *T. brucei s.l.* data were analysed together (Fig. 2A). For both woodlands and plains *T. brucei* data, the inclusion of temporary immunity improved the fit of the model better captured the distinct pattern of intermediate peak and subsequent drop in prevalence with age. Importantly, however, the inclusion of temporary immunity in Model 2 was only statistically significant in the high-transmission woodlands (χ^2^ = 7.537, d.f. = 1, *P* = 0.0.30), but not in the low-transmission plains habitat (χ^2^ = 1.079, d.f. = 1, *P* = 0.299).

None of the *T. b. brucei*-infected samples from lions over 6 yrs of age (n = 21) were co-infected with *T. b. rhodesiense* compared to 18% of *T. b. brucei*-infected lions younger than 6 yrs (n = 50, P = 0.050, Fisher exact 2-tailed test), suggesting that older lions control *T. b. rhodesiense* infection more effectively than younger lions. In the susceptible age group (<6 yrs), 27.8% of plains lions infected with *T. b. brucei* were co-infected with *T. b. rhodesiense*, which is very similar to the 1∶3 ratio of prevalence of *T.b. rhodesiense* to that of *T. b. brucei* consistently observed in all other host species in which the two subspecies co-exist [14]. However, only 12.5% of the *T.b. brucei*-infected woodlands lions were co-infected with *T. b. rhodesiense*, giving a ratio of only 1∶7, and implying that lions subjected to a higher force of infection by *T.b. brucei* are more likely to control *T. b. rhodesiense* infections.

## Discussion

These findings are the first epidemiological evidence consistent with the development of acquired immunity to trypanosomes in any host species. It is unlikely that some hitherto unknown aspect of innate immunity is responsible for the peak and drop in age-prevalence, because this pattern is only seen in *T. brucei* and not *T congolense*. Instead, our observations are consistent with acquired immunity being mounted against *T. brucei*, and several aspects of lion ecology make them ideal candidates for acquired immunity. First, the Serengeti lions are co-infected with multiple species of trypanosomes: besides *T. congolense* (Savannah), *T. brucei* and *T. b. rhodesiense*, lions are also exposed to *T. congolense* (Kilifi and Tsavo), *T. vivax* and *T. simiae*. Trypanosomes switch the variable surface glycoproteins (VSGs) on their surface coats every 3–5 days to avoid immune recognition by the host [15]. Thus on any given day, 6–15 different VSG populations circulate in an infected individual with associated increases in antibody levels [14]. The immune system of a co-infected host is inevitably exposed to a greater variety of VSGs, thus the entire VSG repertoire would be exhausted more quickly if haplotypes were shared across species. The VSG repertoires in the different species and strains circulating within the Serengeti ecosystem are likely to contain many common VSG genes. *T. congolense* s.l. shows far more genetic variability than *T. brucei* s.l. [16], suggesting that the range of naturally expressed VSGs is also higher in *T. congolense* s.l.. *T. brucei*, may in turn generate more variable VSGs than *T. brucei rhodesiense* due to the SRA gene which allows *T. brucei rhodesiense* to avoid lysis in the host bloodstream but which is localised to a single active VSG expression site [17]. Thus, continued high exposure to and increasing infection with *T. congolense* s.l. allows the lions to clear most infections of *T. brucei* s.l. by 3–5 yrs of age and to eliminate all infections of *T. brucei rhodesiense* by the age of 6 yrs. Frequent re-exposure to each trypanosome species by tsetse bites should also accelerate the onset and extent of immunity, thus prevalence should be lowest in areas of high-tsetse exposure (as clearly seen in Fig. 2 for *T. brucei* in the woodlands lions compared to the plains lions, and also the lower ratio of *T. brucei rhodesiense* to *T. brucei brucei* in the woodlands).

Second, carnivores are exposed to trypanosomes through multiple pathways. The plains lions had a similar force of infection for *T. brucei* s.l. as did the woodlands lions, suggesting that infections must also have been acquired by consuming infected meat (since they were exposed to so few tsetse flies). Flesh from infected prey animals would have exposed the lions to as many as 3400 bloodstream-form VSGs in only two years, potentially compromising the predicted repertoire for *T. brucei* [18]–[19]. Thus the acquisition of immunity may be accelerated in any carnivorous host species, but further work will be needed to determine if carnivores generally acquire immunity at younger ages than wild herbivores.

Demonstration of natural immunity is essential if we are to fully understand the epidemiology of human and animal trypanosomiasis and ultimately provide an empirical basis for vaccine development. Recent studies in *T. brucei* have shown that the location of divergence between VSGs is consistent with selection for strain-specific VSG repertoires with the consequence that any vaccine based on large numbers of VSGs from a single strain may only provide partial protection against other strains [20], but such partial protection may be sufficient under natural circumstances to protect animals from harboring human infective *T. b. rhodesiense*. In regions such as E Uganda where cattle act a major reservoir of human infection vaccination would invaluable. Until now, trypanosome researchers have been convinced that immunity is impossible and, hence, that vaccines would be impracticable, but the clear evidence of acquired immunity in lions for *T. brucei* s.l. generates an entirely new perspective on existing data and future research in this field.
